# Cryopreservation Strategies for Poultry Semen: A Comprehensive Review of Techniques and Applications

**DOI:** 10.3390/vetsci12020145

**Published:** 2025-02-08

**Authors:** Areej Arif, Nousheen Zahoor, Jianqiang Tang, Meihui Tang, Liyue Dong, Sardar Zarq Khan, Guojun Dai

**Affiliations:** 1 College of Animal Science and Technology, Yangzhou University, Yangzhou 225000, China; areej.arif.2024@gmail.com (A.A.); nousheenzahoor99@gmail.com (N.Z.); tangjian62538@163.com (J.T.); m19856015485@163.com (M.T.); 13365115757@163.com (L.D.); 2 Riphah College of Veterinary Science, Riphah University, Lahore 05450, Pakistan; zarqniazi22@gmail.com

**Keywords:** semen, cryopreservation, cryoprotectants, poultry, post-thaw, semen-preservation

## Abstract

Poultry semen preservation is crucial for sustaining genetic diversity, improving production efficiency, and facilitating various breeding initiatives. This review underscores the importance and challenges associated with different preservation techniques. We investigate effective techniques for semen collection and evaluation, focusing on vital parameters such as volume, concentration, motility, and morphology. Preservation strategies are categorized into short-term approaches, such as dilution with extenders, and mid-to-long-term strategies, like freezing and the use of cryoprotectants. Additionally, we explore several factors affecting semen quality, including male age and genetics, seasonal impacts, and stress during handling. Assessing the quality of preserved semen is critical, particularly regarding post-thaw motility. The applications of these preservation techniques in artificial insemination, genetic enhancement, the conservation of endangered breeds, and biobanking are highlighted. This review identifies critical research opportunities, including the development of improved cryoprotectants, refining freezing protocols, comprehending the mechanisms of semen damage, and innovating novel preservation technologies. Addressing these challenges will enhance poultry semen preservation and contribute to sustainable poultry farming.

## Simple Summary

This review highlights the significance of poultry semen preservation for enhancing genetic diversity and breeding efficiency. It categorizes preservation techniques into short-term (using extenders) and long-term (freezing with cryoprotectants), addressing factors that impact semen quality, such as male age and handling stress. Effective semen collection methods and quality evaluation are essential for successful artificial insemination. Current research focuses on optimizing cryopreservation techniques to improve sperm viability and fertility, contributing to sustainable poultry farming practices.

## 1. Introduction

The development of artificial insemination (AI) techniques, which originated in Russia, gained significant momentum in Europe and the United States between 1933 and 1938 [1]. By 1953, the need for long-term sperm storage led to the advent of bull sperm cryopreservation [2]. A landmark discovery came with the accidental identification of glycerol as a cryoprotectant (CPA) over seven decades ago, enabling the first successful cryopreservation of chicken semen and laying the foundation for poultry cryobiology [3]. Despite these advancements, poultry semen cryopreservation faces significant challenges, such as inconsistent fertility rates following freezing and thawing [4,5,6]. The unique reproductive physiology of avian species further complicates the preservation of embryos and oocytes, making semen cryopreservation essential for poultry AI. Nearly 95% of AI procedures in poultry rely on preserved (or diluted) semen rather than fresh semen [7]. Effective semen preservation requires optimized media to extend the lifespan of spermatozoa by halting or interrupting their metabolic activity [7,8,9]. Spermatozoa are highly specialized cells designed to deliver paternal DNA and activate the egg during fertilization. Despite this shared function, sperm characteristics vary significantly among species, including differences in head morphology, movement patterns, plasma membrane composition, and freezing tolerance. For instance, poultry sperm, particularly those of the *Galliformes* order, possess a unique thread-like structure with narrower and longer sperm heads, resulting in a smaller overall cell volume compared to mammalian sperm [10]. Notably, the fertilizing ability of cryopreserved rooster semen retains less than 2% of the fertilizing capacity of fresh semen [11].

Among the approximately 10,000 known avian species, 13% are considered imperiled, equating to nearly 1375 species at significant risk of extinction [12]. According to FAO statistics, 19% of the world’s 1641 chicken breeds are endangered, vulnerable, or extinct, with the proportion of avian breeds at unknown risk notably higher than mammals (64% vs. 59%) [13]. This alarming statistic highlights the urgent need for conservation strategies aimed at preserving genetic diversity and protecting endangered avian species. In the poultry industry, chickens are the most abundant domesticated animals. Annually, 60 billion chickens are raised, producing approximately 100 million tons of meat and 70 million tons of eggs for global consumption [14]. Generations of selection for desirable traits and inbreeding have led to a 50% reduction in genetic diversity in commercially pure lines [15]. Chicken biodiversity faces significant threats due to the widespread production of commercial hybrid chickens in industrial systems. The rapid decline in poultry genetic resources poses a significant threat to livelihoods.

**Figure 1:**
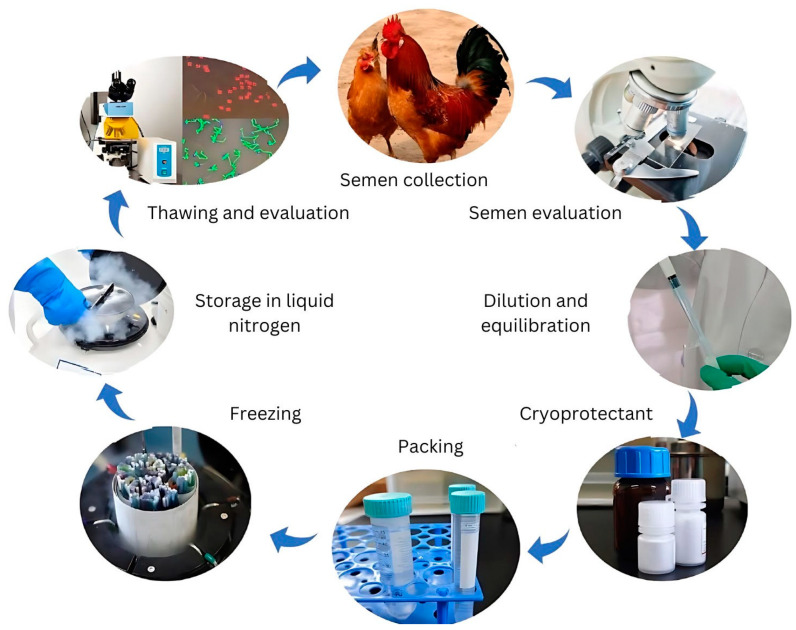
Key processes of chicken sperm cryopreservation technology.

Efforts to preserve genetic resources have focused on maintaining live populations and developing cryopreservation technologies. However, live populations remain vulnerable to biodiversity loss caused by population fluctuations, deleterious inbreeding, infectious disease outbreaks, and environmental disasters [16,17]. Cryopreservation has thus been recognized as a crucial tool for managing genetic diversity and conserving endangered breeds. While chicken sperm cryopreservation has been studied for over six decades, a standardized protocol applicable to all breeds and lines remains elusive. Various factors—including semen extenders, cryoprotectants, pre-freezing manipulations, packaging, and freezing–thawing rates—significantly influence cryopreservation efficiency, as illustrated in Figure 1.

## 2. Semen Collection and Evaluation

### 2.1. Pre-Collection Preparations

Before semen collection, it is essential to gather a detailed history of the male bird [18,19,20]. This includes the breeding history, medical history, a record of medications and supplements taken within the past 6 months, genetic and familial information, and an assessment of the degree of inbreeding. Additionally, the time since the last breeding or collection should also be recorded [21].

### 2.2. Collection Methods

#### 2.2.1. Manual Collection (Massage Method)

The development of manual collection techniques was driven by the desire to improve genetic selection and enhance breeding efficiency. Initially, these methods were simple and required restraining the male birds to collect semen. As knowledge of avian reproductive physiology advanced, these techniques were refined, resulting in higher semen quality and quantity [22,23]. One significant adaptation, introduced by Gabriel [24], involved a confined elongated cone for rooster semen collection. The cone is placed at a slight angle, allowing the rooster to remain calm while exposing the abdomen and legs for semen extraction. The massage technique, developed by Burrows et al. [25], stimulates ejaculation, which can vary in semen volume from 0.05 to 0.50 mL in light breeds to 0.1–0.9 mL in heavy breeds [26]. This method marked a shift from older practices, such as euthanizing hens to retrieve semen from the oviduct, demonstrating its ethical and practical advantages [27].

#### 2.2.2. Modern Collection

Modern semen collection methods in poultry have advanced to include electroejaculation and hormonal stimulation, both of which offer significant improvements over traditional methods.

- Electroejaculation: Electroejaculation involves the use of mild electrical pulses to stimulate ejaculation in male birds. This technique is particularly useful when manual collection methods are ineffective or when a male bird is unresponsive to traditional collection methods. Studies, such as the one conducted by Santiago-Moreno et al. [28], have demonstrated the successful application of electroejaculation in galliform birds, including quail and pheasants. Electroejaculation has become an essential tool in both research and conservation settings, allowing for the collection and preservation of semen from valuable males without the need for natural mating. It is also a key technique in breeding programs where traditional methods may fail.
- Hormonal stimulation: Hormonal treatments can be used to stimulate semen production in males that are not responsive to conventional methods. This method typically involves the use of hormones like gonadotropins to enhance sperm production and induce ejaculation. Hormonal stimulation has been explored in poultry to improve the consistency and volume of semen collection, particularly in males with low sperm production [29].

### 2.3. Frequency of Semen Collection

Studies showed the frequency of semen collection in broiler breeder-type and Leghorn males, yielding notable results. Broiler breeder-type males ejaculated three times a week, producing larger semen volumes and a higher sperm cell count compared to other frequencies. Similarly, Leghorn males collected three times weekly also exhibited significantly increased semen volume and higher concentrations of sperm cells. Those collected once weekly had sperm concentrations similar to the three-times group but produced more sperm cells than those collected five times weekly. Overall, the frequency of collection did not impact fertility in either type [30]. The findings indicated that the frequency of semen collection affected semen volume and sperm cell numbers but had no bearing on fertility.

1. Variation by species: The optimal frequency for semen collection differs across poultry species. Chickens may require collection every 2–3 days, whereas turkeys maybe collected once a week [31].
2. Reproductive phase: Semen collection often coincides with the bird’s reproductive phases especially during peak breeding times, when collection may be more frequent [31].
3. Purpose-driven collection: The intended use of semen, whether for AI or research purposes, influences the frequency of collection. Adjustments are often made to meet specific breeding goals [32].
4. Health and welfare issues: it is essential to ensure that the collection frequency does not adversely affect the bird’s health, as frequent collection may impact this [32].
5. Optimal practices: adhering to established protocols [31] during semen collection is vital for preserving semen quality and optimize fertility rates in breeding initiatives.

The efficiency of semen collection greatly influences the success of AI, and ensuring a sufficient number of high-quality sperm is critical for achieving optimal fertility rates [33,34]. Once the appropriate collection frequency is established, the next critical step is evaluating the semen’s quality, ensuring its suitability for breeding purposes.

### 2.4. Evaluation of Semen Quality

Semen quality is a paramount determinant of both the fertility and hatchability of eggs in poultry [35]. Evaluating semen quality serves two main purposes, primarily to ensure that males producing good quality semen are retained and to measure spermatozoa concentration and semen volumes. The number of viable sperm per dose depends on insemination decisions [36]. The next sections describe an integrated assessment procedure, starting with macroscopic and then proceeding through microscopic tests to biochemical analysis.

#### 2.4.1. Semen Appearance (Macroscopic Evaluation)

Visual inspection is still a necessary first step in semen evaluation. Good quality semen is thick and pearl white; any deviations such as yellow, green, or reddish coloration may indicate contamination, injury, or infection [37,38,39]. This preliminary visual examination helps identify potential issues that can be further investigated through more detailed microscopic and biochemical methods.

#### 2.4.2. Sperm Motility Assessment

Sperm motility, a critical aspect of microscopic evaluation, is a key indicator of semen quality, reflecting the proportion of viable sperm in a sample. It is crucial for assessing the fertilizing potential of semen. Typically, sperm motility is evaluated by examining fresh and diluted semen under a light microscope at 100× magnification. A motility rate of ≥ 70% is considered optimal for successful fertilization [40].

Traditional methods are as follows:

- Light microscopy: Light microscopy is a widely used method where sperm cells are directly observed, providing a qualitative measure of motility. However, this approach is subjective and lacks a detailed analysis of sperm movement characteristics [40].
- Manual sperm motility scoring: manual sperm motility scoring classifies sperm as rapid, slow, or non-motile based on visual assessment, a technique that, while inexpensive, relies heavily on the technician’s experience.
- Viscosity test: the viscosity test evaluates the sperm’s ability to move through fluids of varying viscosities, simulating the challenges sperm face in the reproductive tract.

Advanced methods are as follows:

- Computer-assisted semen analysis (CASA): CASA utilizes advanced imaging and analytical software (Sperm Class Analyzer (SCA 6.5)) to automate the assessment of sperm parameters such as motility, concentration, and morphology [41]. It quantifies sperm movement using metrics like straight-line velocity (VSL), curvilinear velocity (VCL), and average path velocity (VAP). Sperm are categorized as slow (<10 μm/s), medium (10–50 μm/s), or rapid (>50 μm/s), providing a precise evaluation of their fertilization potential. The method’s high accuracy and reproducibility make it invaluable for research and breeding programs [42].
- Flow cytometry: This method assesses sperm motility, viability, concentration, and DNA integrity by analyzing light scatter and fluorescence. For example, light scatter indicates cell size and structure, while fluorescence reveals membrane integrity or DNA fragmentation. Flow cytometry (FlowJo (10.8)) enables rapid high-throughput analysis of sperm quality [43].
- Digital image analysis (DIA): DIA captures high-resolution images of sperm and uses specialized software (ImageJ (1.53) for quantitative motility assessments. Unlike manual scoring, it minimizes human error and provides detailed morphological and motility data, making it ideal for large-scale evaluations.
- High-resolution video microscopy: This technique records sperm movement in high definition at elevated frame rates, enabling the detection of subtle motility defects and abnormal behaviors. For instance, it can identify irregular flagellar motion or hyperactivated motility patterns, offering deeper insights into reproductive performance. Combining traditional and advanced techniques enhances the reliability of semen evaluation. For instance, integrating CASA with manual motility scoring ensures a comprehensive assessment: CASA provides precise quantitative data, while manual scoring offers practical insights in resource-limited settings. Studies have shown significant correlations between sperm concentration, motility, and morphology, further emphasizing the value of combining these methods [42].

#### 2.4.3. Sperm Morphology

**Figure 2:**
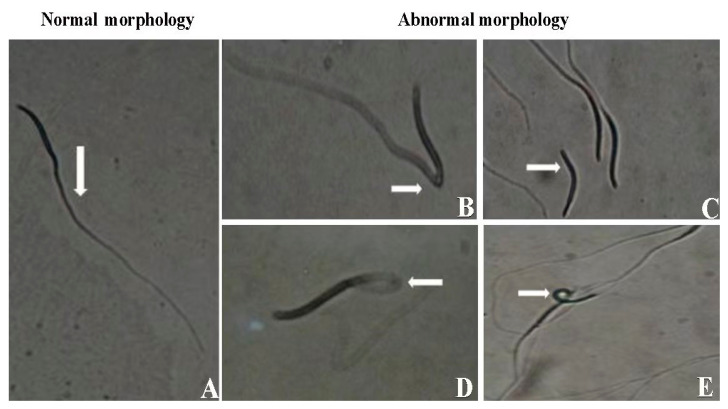
Different types of sperm observed during morphological evaluation. (**A**) Morphologically normal. (**B**) Bent at midpiece. (**C**) Coiled head. (**D**) Broken tail. (**E**) Loose head reprinted and modified with permission from [46].

The morphology of sperm is a critical indicator of semen quality, offering valuable insights into sperm health and viability. For optimal fertility outcomes, a minimum threshold of 80% normal sperm morphology is typically required [44]. However, other factors such as motility, membrane integrity, and DNA quality are also essential in ensuring the sperm’s functionality and fertility potential. The eosin–nigrosine staining technique developed in [44] is widely used in avian sperm morphology assessment. This method distinguishes live from dead sperm, with viable sperm excluding the stain. While normal sperm morphology is important for fertility, the sperm’s ability to ascend the hen’s reproductive tract and reach the sperm storage tubules (SSTs) also depends on motility, metabolic activity, and interactions with the female reproductive environment [45]. Figure 2 represents different types of sperm observed during morphological evaluation.

#### 2.4.4. Biochemical Tests

Semen evaluation includes a variety of physical and biochemical tests [47,48,49,50]. Common tests include assessments of pH, fructose levels, and enzymatic activity. Optimal pH levels range from 7.2 to 7.8 for poultry semen.

## 3. Semen Preservation Techniques

The scientific exploration of semen preservation began in the 18th century, highlighted by Spallanzani’s observation of stallion spermatozoa frozen in snow. This discovery demonstrated that sperm could enter a state of suspended animation, paving the way for subsequent advancements in sperm viability research. This breakthrough led to significant progress in cryopreservation, particularly with the use of glycerol as a cryoprotectant, which revolutionized low-temperature preservation techniques across various species [51].

While semen freezing technology has yielded notable success in many species, avian semen preservation remains a significant challenge. Initial attempts to freeze chicken semen have not produced satisfactory results, underscoring the need for more advanced preservation methods. Cryopreservation, defined as the freezing of sperm at −196 °C in liquid nitrogen, is essential in maintaining sperm viability. For poultry, cryopreservation plays a vital role not only in production but also in conservation efforts, as preserving embryos and oocytes in female birds remains difficult. The successful cryopreservation of avian sperm is crucial for conserving endangered species, maintaining genetic diversity in commercial poultry breeds, and improving genetic material preservation. Although some techniques have shown promising sperm vitality and fertilization rates, further advancements are necessary to improve long-term viability and fertility [44]. The first successful preservation of chicken semen was achieved in 1949 [3]. Sperm freezing involves several critical steps, including selecting a suitable male, a semen quality assessment, dilution, cryoprotectant addition, packaging, freezing, cryoprotectant removal, and post-thaw sperm viability evaluation [52,53].

### 3.1. Short-Term Preservation

The short-term preservation of poultry semen typically lasts up to one week, and several challenges hinder its effectiveness, including bacterial contamination and suboptimal conditions by incorporating antioxidants to reduce oxidative stress and antimicrobials to prevent bacterial growth. Additionally, optimizing dilution ratios and storage conditions plays a vital role in enhancing sperm viability during short-term preservation.

#### 3.1.1. Dilution with Extenders and Cryoprotectants

A rooster typically ejaculates 0.2–0.7 mL of semen, with sperm concentrations ranging from two to seven billion per ml. To preserve semen effectively, an ideal extender must provide energy to sustain extender formulations. Research has focused on improving extenders’ sperm metabolism, maintain an optimal pH, and ensure appropriate osmolality, thereby supporting sperm viability and functionality [54,55,56]. Extenders contain a combination of cryoprotectants and nutrients, which protect sperm from freezing-induced damage, osmotic stress, and other detrimental factors.

Role of cryoprotectants: Cryoprotective agents (CPAs) are crucial in protecting sperm from damage during freezing and thawing. These agents are classified as internal or external based on their mechanisms of action.

Internal cryoprotectants: these agents penetrate sperm cells and prevent intracellular ice formation while stabilizing cellular structures.

- Glycerol is used in extenders like the Lake PC extender to prevent ice formation and stabilize sperm membranes.
- Dimethylformamide (DMF) is utilized in the BHSV extender to lower the freezing point and protect against osmotic stress.
- Dimethylacetamide (DMA) is found in the FEB extender to reduce ice formation and osmotic damage during freezing.
- Ethylene glycol (EG) is added to several extenders to penetrate sperm membranes and prevent intracellular ice formation.
- Propylene glycol (PG) stabilizes sperm membranes and reduces damage during freezing.
- Dimethyl sulfoxide (DMSO) protects sperm by penetrating the cell membrane and preventing ice formation.
- *Trehalose* stabilizes proteins and lipids during freezing, enhancing sperm membrane protection.

External cryoprotectants: these agents protect sperm externally, mainly by combating oxidative stress and membrane damage.

- Egg yolk provides protection due to its high lipid content, stabilizing sperm membranes during freezing and thawing.
- Polyvinylpyrrolidone (PVP) enhances sperm motility and protects membranes during freezing.
- Myo-inositol is an antioxidant that reduces oxidative damage during freezing and thawing.
- Citrate maintains sperm pH and reduces oxidative stress, enhancing sperm quality.
- Vitamins (e.g., Vitamin E and C) are antioxidants that neutralize free radicals, protecting sperm from oxidative damage.
- Formamide is an external CPA that has proven effective in some species for protecting sperm during cryopreservation.

The composition of extenders, which contains these cryoprotectants, is key to effective semen preservation. For example, egg yolk-based extenders offer significant protection against cold shock by stabilizing sperm membranes.

#### 3.1.2. Importance of Dilution

Dilution is essential in poultry semen cryopreservation, as it helps prevent semen deterioration and maintains sperm viability during both freezing and thawing. By reducing the concentration of metabolic byproducts and minimizing substrate depletion, dilution ensures a stable environment for sperm cells. It also mitigates osmotic shock and ice crystal formation, which can damage sperm cell membranes and reduce fertility post-thaw. Proper dilution techniques support sperm metabolism, preserve cellular integrity, and enhance fertilization and hatching rates in poultry breeding programs [57,58]. Reducing substrate depletion ensures that sperm remain viable and capable of fertilizing eggs, ultimately improving hatching rates in poultry [59,60].

The composition of extenders used for dilution is critical to sperm preservation. Egg yolk-based extenders, rich in lipids, provide significant protection against cold shock by stabilizing sperm membranes. Cryoprotectants, such as glycerol, further enhance survival rates by preventing ice crystal formation and reducing osmotic stress during the freezing process [61]. This combination helps maintain sperm viability and functionality, contributing to favorable fertility outcomes and high hatching rates in poultry breeding programs [58].

#### 3.1.3. Limitations of Short-Term Preservation

The effectiveness of short-term poultry semen preservation typically lasts about one week but presents several limitations. A significant issue is the risk of bacterial contamination if extenders are not properly formulated. This can lead to a decline in sperm quality and motility over time, ultimately affecting the success of AI. Current research efforts focus on improving extender formulations to enhance poultry semen preservation. The improvements include adding antioxidants to mitigate oxidative stress on sperm cells, which enhances their viability, and using antimicrobials to prevent bacterial contamination that could compromise sperm quality. Researchers are also examining the effects of various dilution ratios and storage conditions on sperm viability to identify optimal strategies for effective short-term preservation.

#### 3.1.4. Extenders in Current Use

Several extenders are commonly used for poultry semen cryopreservation. Traditional extenders such as Ravie and Lake extenders are frequently utilized in avian practices [62]. The BHSV [63] extender is another widely used option, recognized for its efficacy in preserving semen quality, while the Beltsville [64] is used less. Additionally, the EK extender [65] is well known for its ability to maintain sperm viability during preservation. Recent studies have identified additional extenders that show promise. Poultry Media has been recognized as a suitable extender, often supplemented with cryoprotectants to enhance its performance. Raptac is effective in maintaining sperm motility post-thawing, though NeXcell is less effective compared to Poultry Media and Raptac but still used in specific conditions. Furthermore, N-methylacetamide (NMA), although not an extender itself, is frequently incorporated into existing extenders to improve sperm viability during cryopreservation [54]

The costs associated with these extenders are influenced by factors such as cryoprotectants, freezing straws, liquid nitrogen, and labor, with overall sample preparation expenses typically ranging from USD 20 to USD 50. However, these costs can fluctuate depending on geographical location and supplier pricing, impacting the overall expense of cryopreservation efforts [66].

#### 3.1.5. Recommended Dilution Rates

A dilution rate ranging from 1:1 to 1:4 is typically recommended for chicken semen freezing [67,68]. However, studies have shown that this dilution rate does not significantly affect fertility outcomes. Research indicates that within this range, fertility outcomes remain unaffected when appropriate cryoprotectants and extenders are employed [60]. For instance, studies on breeds such as the White Leghorn have demonstrated fertility rates exceeding 88%, particularly when extenders containing egg yolk and glycerol are utilized [69]. Interestingly, certain studies suggest that lower dilution ratios, such as 1:2, can achieve satisfactory post-thaw motility and fertilizing ability without compromising sperm quality [70]. In some cases, dilution ratios outside the conventional 1:1 to 1:4 range—such as 1:5 or 1:10—have been reported to maintain acceptable post-thaw motility and fertility, as long as the extender composition is properly optimized. However, it is important to note that the success of semen cryopreservation is not solely dependent on dilution rates. Other factors, including the composition of the extender, as well as the freezing and thawing protocols, also play a crucial role in preserving sperm quality, maintaining motility, and ensuring successful fertility outcomes [60].

#### 3.1.6. Research Gaps and Future Directions

Systematic research on the effects of extender parameters on chicken sperm is essential for advancing both fresh and frozen sperm viability and fertility [71]. Different extenders are often formulated with specific internal cryoprotective agents (CPAs) tailored to optimize cryoprotection and improve sperm cell survival. However, there is a need for comprehensive studies to better understand how various extender components, such as cryoprotectants, antioxidants, and energy substrates, interact to influence sperm quality during storage and post-thaw recovery [72]. Future research should also focus on optimizing extender formulations for different poultry breeds, as variations in genetic and physiological traits may impact sperm response to cryopreservation. Investigating novel CPAs and alternative cryopreservation techniques, such as vitrification or nano-technology-based approaches, could further improve sperm viability and fertility rates [73]. These efforts are essential for advancing poultry breeding programs and ensuring their sustainability.

#### 3.1.7. Procedure Overview

Various methods and techniques were already discussed above. Briefly, the procedure was as follows:

- Gently mixing the semen with the extender.
- Cooling the mixture to 4 °C for 15 min.
- The extender containing the internal cryoprotective agents (11% glycerol, 6% DMA, or 6% DMF) was also equilibrated at 4 °C.
- The CPA-enriched extender was added to the semen samples and allowed to equilibrate at 4 °C.
- Semen was subsequently transferred into sealed 0.5 mL plastic freezing straws and then placed in a biological freezer unit and frozen using the controlled cooling rates detailed for each experiment and treatment in Table 1 before being plunged into liquid nitrogen. All of the following experiments on dilution rate, MET assay, insemination dose, storage duration, and R+ family restoration relied on glycerol-based freezing protocol.

#### 3.1.8. Pricing Information

The costs associated with poultry semen cryopreservation vary based on several factors, including cryoprotectants (USD 50–USD 150 per 100 mL), freezing straws (USD 0.20–USD 0.50 each), liquid nitrogen (USD 1.00–USD 3.00 per liter), and labor and equipment (USD 20–USD 50 per experiment). Consequently, the estimated cost per sample ranges from USD 20 to USD 50, depending on geographical location, supplier pricing, and laboratory practices [74].

**Table 1:** Different cryoprotectants and their methods of use for rare breed semen storage [75].

Internal Cryoprotectant	GLY	EG	DMF	DMA
**Extender**	Lake PC	BHSV	BHSV	FEB
**External cryoprotectant**	PVP	Myo-inositol	Myo-inositol	PVP
**Semen collection**	1 mL semen in 1 mL extender	1 mL semen in 1 mL extender	1 mL semen in 1 mL extender	1 mL semen in 1 mL extender
**Cooling**	4 °C, 15 min	4 °C, 15 min	4 °C, 15 min	4 °C, 15 min
**% Internal CPA**	11%	10%	6%	6%
**CPA addition**	In 1 mL extender	In 1 mL extender	In 1 mL extender	In 1 mL extender
**Equilibriation time**	10 min	10 min	4 min	2 min
**Freezing rate**	−7 °C/min	−1/min	−15 °C/min	−60 °C/min
**Thawing (°C -min)**	4 °C, 3 min	4 °C, 3 min	4 °C, 3 min	40 °C, 5 s
**Glycerol removal**	Dilution 1/20, 550 g 4 °C—15 min	No glycerol removal	No glycerol removal	No glycerol removal
**No. of female/treatment**	20		20	20
**IA dose**	400 × 10^6^ spz	400 × 10^6^ spz	400 × 10^6^ spz	400 × 10^6^ spz
**No. of AI**	5	5	5	5
**Frequency of AI**	Every day 4 d	Every day 4 d	Every day 4 d	Every day 4 d
**Egg collection**	J2 to J5	J2 to J5	J2 to J5	J2 to J5
**Cost per 100 mL Cryoprotectant**	USD 50–USD 150	USD 50–USD 150	USD 50–USD 150	USD 50–USD 150
**Cost per freezing straw**	USD 0.20–USD 0.50	USD 0.20–USD 0.50	USD 0.20–USD 0.50	USD 0.20–USD 0.50
**Cost per liter of liquid nitrogen**	USD 1.00–USD 3.00	USD 1.00–USD 3.00	USD 1.00–USD 3.00	USD 1.00–USD 3.00
**Labor and equipment costs per experiment**	USD 20–USD 50	USD 20–USD 50	USD 20–USD 50	USD 20–USD 50
**Estimated cost per sample**	USD 20–USD 50	USD 20–USD 50	USD 20–USD 50	USD 20–USD 50

#### 3.1.9. Specific Breeds of Hens

Cryopreservation techniques were applied to several breeds of hens, each selected for their unique characteristics and reproductive performance. Notably, the two following breeds were emphasized:

- White Leghorn: Renowned for its high fertility rates, this breed is commonly used in commercial egg production. Its reproductive efficiency makes it a prime candidate for cryopreservation studies, where maintaining high post-thaw fertility is essential.
- Rhode Island Red: Valued for its hardiness and adaptability, this breed is capable of thriving in various environmental conditions. Research indicates that its sperm may exhibit different viability characteristics during cryopreservation, making it an important breed to consider in the optimization of freezing protocols [44].
- Understanding the specific traits of these breeds is crucial for enhancing the effectiveness of cryopreservation techniques and ensuring successful fertility outcomes in poultry breeding programs.

### 3.2. Mid-Term Preservation

When semen requires preservation for longer than 30 min up to 48 h, it must be diluted in isotonic solution with a neutral pH to prevent the rapid depletion of freezing capacity. Avian sperm cells are devoid of energy reserves, which is an important factor to consider during in vitro storage. Furthermore, their metabolism produces reactive oxygen species (ROS), and the acrosome reaction can occur without any specific induction process. Avian seminal plasma plays a pivotal role in stimulating sperm motility, regulating membrane stability, and supporting the fertilization process. However, it also contains fractions that are detrimental to sperm storage, including ROS, and various proteolytic and lipolytic enzymes [76]. The diluent generally contains antibiotics like penicillin, streptomycin, or gentamicin to prevent bacterial contamination during storage [77]. However, there is a growing interest in finding alternative methods because of the problems of antibiotic resistance and possible residues. Certain recent investigations have documented that natural compounds, including those from plants or peptide-based inhibitors, may serve as promising alternatives to antibiotics in semen diluent, although further studies are needed for practical applications [78]. Elevated metabolism and enzymatic activity in the seminal plasma of avian sperm necessitate its rapid dilution with neutral diluent, including fructose- or glucose-enriched diluent, occasionally supplemented with antioxidants and zwitterion buffers to prevent it from rapid degradation [76]. Undiluted fresh chicken sperm forfeits its fertilizing ability in less than an hour [6]. Chilling fresh semen from 41 °C to a temperature of 20 °C is an essential step for storing it for less than 1 h. This temperature reduction mitigates the activity of semen enzymes and sperm cell metabolism, consequently diminishing the production of acetic acid and reactive oxygen species (ROS) [79]. Studies have reported that sperm viability was satisfactorily maintained for 24 h of refrigeration in sodium glutamate–polyvinylpyrrolidone-based (L&R-84) medium [62]. At 48 h of refrigeration, the Lake 7.1 medium was shown to have better performance due to the inclusion of zwitterion buffer (BES) (Table 2) [80]. This helps mitigate pH fluctuations during the cooling process. The osmolarity of chicken seminal plasma is known to range from 320 to 330 mOsm/kg. Notably, some extenders have osmolarities exceeding the physiological range of rooster sperm, and recent unpublished data suggest that this may negatively affect sperm motility. Accordingly, the use of extenders with an osmolality similar to that of the seminal plasma is advised. Nevertheless, this recommendation should be applied with caution, as a decrease in motility during storage could be advantageous in reducing ROS production provided the reduction in motility is reversible and there is no disruption of cell membranes.

#### Conventional and Directional Freezing Techniques

The star-shaped ice crystal freezing method is a manual technique [81] that involves exposing semen to liquid nitrogen vapor at a height of 4–5 cm for 10–15 min, achieving a freezing rate of approximately 60 °C/min [81,82,83,84]. However, conventional freezing techniques vary among species, depending on sperm quality and cryopreservation survival post-thawing [84]. Both rapid freezing and slow-controlled freezing, using either liquid nitrogen or vapor-phase nitrogen, can preserve sperm integrity. This suggests that the choice of freezing method may not be as crucial as once thought, offering more flexibility in cryopreservation techniques [85]. The directional freezing technique employs a multi-thermal gradient approach [82,83], offering a controlled and precise method for cryopreservation. First, semen is diluted with a freezing extender and chilled to 4 °C to 5 °C at a rate of 0.3 °C/min. The semen is then packaged in pre-chilled tubes and moved through a linear temperature gradient from 5 °C to 50 °C at constant velocity of 1 mm/s. After seeding, the tubes are transferred to a collection chamber at −100 °C, where the freezing process is optimized before final storage in liquid nitrogen [81,86].

The preservation of poultry semen for the medium term primarily involves two methods, namely conventional freezing and directional freezing. Conventional freezing cools sperm in extenders prior to freezing them at −196 °C. This method faces considerable challenges, such as fluctuating post-thaw motility and viability. Upcoming research mainly focuses on identifying optimal cryoprotectant levels to improve survival rates, exploring the effects on post-thaw performance, and minimizing cellular damage. Directional freezing is a precise technique designed to manage heat removal and minimize ice crystal formation, which can damage sperm cells. However, this method presents challenges, such as a complex setup and the need for the precise control of environmental conditions. Upcoming research will primarily focus on its impact on sperm morphology and functionality, as well as developing portable devices to facilitate its use in poultry reproduction.

### 3.3. Long-Term Preservation

#### 3.3.1. CPAs Preservation Technique

CPAs play a key role in preserving sperm by protecting it from the damaging effects of ice crystal formation, chemical exposure, and osmotic stress. Their mechanism of action involves increasing solute concentration, which reduces ice formation. This regulates the rate of cell dehydration during freezing, minimizing the likelihood of intracellular ice formation [87]. While glycerol is a standard cryoprotectant in poultry semen storage, it has been shown to negatively affect sperm motility when introduced into the female reproductive tract, creating a trade-off between its ability to preserve semen and its fertilization capacity [88]. A concentration of 8–11% glycerol is widely used for this purpose [89]. Other commonly used cryoprotectants include DMSO (dimethyl sulfoxide) and propylene glycol, with concentrations ranging from 5% to 15% which are effective in achieving the best outcomes [90]. Good fertility has been achieved with chicken semen frozen using dimethylacetamide (DMA), especially in pellets formed by plunging 50-microliter droplets of semen directly into nitrogen [91]. This method provides a rapid cooling rate of approximately 600 °C/min. However, plastic straws have become the most widely used packing method for cryopreservation programs, owing to their ease of use and identification. Interestingly, when freezing chicken semen in straws, the best post-thaw quality is typically observed with cooling rates between 50 °C and 250 °C. A widely endorsed two-stage cooling protocol involves decreasing the temperature from 5 °C to −35 °C at a rate of 7 °C/min, followed by a further reduction from −35 °C to −140 °C at a rate of 60 °C/min [92]. It is not essential to eliminate dimethylformamide (DMF) before insemination, and studies have shown that satisfactory fertility outcomes can still be achieved even with DMF present [93,94].

The cryotolerance of avian sperm may be influenced by the amino acid profile of the seminal plasma. For example, valine has been shown to contribute to the preservation of DNA integrity and enhance the viability of frozen-thawed rooster sperm [76]. The most pronounced effects are observed in breeds whose sperm are least resistant to cryopreservation [95]. Numerous studies have also explored additives, such as antioxidants or plant-derived compounds with antioxidant properties (e.g., resveratrol, lycopene, quercetin), to improve the cryoprotective capacity of semen extenders [96]. CPAs can be toxic to sperm at higher concentrations, and their efficacy varies across species. Ongoing research aims to identify non-toxic CPAs that promote sperm viability and determine the best combinations to improve cryopreservation outcomes, ultimately improving the preservation of poultry semen.

#### 3.3.2. Sperm Vitrification Techniques

Vitrification is a rapid cooling process that turns the solution into a glassy state, preventing the formation of ice crystals [97]. This technique relies on high cooling rates, viscosity, and small volumes, making it suitable for sperm preservation [98]. Promising results have shown high post-thaw quality, underscoring its potential for fertility preservation. A typical vitrification solution is prepared using N-2-hydroxyethylpiperazine-N-ethane sulfonic acid (HEPES)-buffered medium 199 and 20% calf bull serum [99]. This process requires precise handling and expertise, and its effectiveness may vary depending on the sperm species or strain.

Recent advancements have highlighted the use of innovative cryoprotectants, resulting in improved sperm motility and DNA integrity compared to conventional freezing methodologies [100]. Moreover, ongoing research into the long-term viability of avian sperm underscores the urgent need for optimized vitrification protocols to enhance sperm preservation efficacy [73]. Comparative studies indicate that vitrification surpasses conventional freezing methods in sperm preservation, offering superior post-thaw motility and reduced DNA fragmentation, thus making it a better option for clinical applications [101]. Despite its benefits, vitrification requires expert handling, and its efficacy may vary across species. Current research focuses on optimizing vitrification techniques for various species and investigating the mechanism of cryoinjury and the influence of cryoprotectants on sperm functionality [101].

#### 3.3.3. Primordial Germ Cell Isolation and Cryopreservation

PGCs are the precursor cells that give rise to ova and spermatozoa. They are detectable in the gonads of young chicken embryos prior to the sixth day of incubation. The use of this technique has been successful in chickens, but studies on quail are still in progress [102]. PGCs have applications in genetic conservation transgenesis in chickens. PGCs are cultured in vitro, cryopreserved, and then transplanted into host embryos, where they mature into gametes, aiding in the preservation of genetic resources [80]. This process is complicated and presents challenges, especially regarding the long-term survival and functionality of PGCs after thawing. Ongoing studies focus on improving PGCs and investigating their capacity to regenerate sperm in vitro, which could enhance poultry reproductive technologies.

Recent research focuses on several key areas to enhance the understanding and application of PGCs in poultry reproductive technologies as follows:

- Optimization of Cryopreservation Techniques

Researchers are exploring different cryoprotectants and freezing techniques to boost the viability of cryopreserved PGCs. Results show that different cryoprotectants can significantly influence PGC motility and DNA integrity, indicating the need for tailored methods depending on the species and cell type [103].

- Mechanism of Cryoinjury

Understanding the mechanisms of cryoinjury is important for preserving PGC viability. Ongoing research examines how different cryoprotectants influence PGC viability and functionality after thawing, potentially leading to better preservation techniques.

- In Vitro Regeneration of Sperm

Studies are focused on enhancing PGCs ability to regenerate sperm in vitro. This could significantly impact poultry reproduction by facilitating the production of viable sperm from cryopreserved PGCs, helping maintain genetic diversity and improve breeding programs [104].

- Transgenic Applications

Researchers are exploring the potential of PGCs in transgenic applications. By modifying these cells before transplantation, they aim to produce chickens with traits like enhanced growth and disease resistance. This research has the potential to enhance poultry farming [105,106].

- Long-Term Viability Studies

Current research is examining the long-term viability of PGCs after thawing. Researchers are exploring the effects of different culture conditions and freezing techniques on the long-term functionality of PGCs, which is essential for their use in breeding programs [104,107].

Research on PGCs in chicken embryos is essential for boosting poultry reproductive technologies and conserving genetic diversity in poultry.

#### 3.3.4. Gonad Cryopreservation and Transplantation

The process of gonad cryopreservation and transplantation usually begins with the extraction of gonads from newborn poultry. These gonads are stored in a specialized solution (DPBS-FBS) or kept on ice until they can be frozen or implanted. It is essential to ensure that the removed gonads are free from any additional tissues, as this facilitates proper placement and reduces scarring, ultimately improving graft performance. Nevertheless, numerous ethical issues and regulatory hurdles complicate this procedure. Additionally, there is a risk of tissue damage during freezing and transplantation. Ongoing research focuses on refining surgical techniques and understanding the immune system’s response to these transplants. This research ultimately enhance the effectiveness of fertility restoration procedures [80,108,109].

## 4. Factors Affecting Semen Preservation

The collection of semen and the factors that influence its quality are critical. Gentle collection techniques, consistent timing, and strict hygiene protocols are essential to prevent sperm damage or contamination. These factors play a crucial role in preserving the integrity of the sperm for future use. An extender serves as a protective medium for the sperm cells. Selecting a suitable extender that provides protection, essential nutrients, and osmotic balance is crucial; every step is important. Storage conditions must be kept at the correct temperature. Furthermore, the environment in which roosters are kept significantly influences sperm viability. Excessive light, extreme temperatures, and stressful conditions can all negatively impact sperm health. Cryopreservation negatively impacts the viability and survival of the thawed sperm, attributed to the disruption of the functional integrity of the sperm’s acrosome, plasma membrane, DNA, and mitochondria incurred during the freezing procedures [110,111]. The mitochondrial membrane of fresh sperm exhibits a dense uniform matrix. Freezing and subsequent thawing leads to a decrease in the density of the mitochondrial matrix and results in subtle swelling of the mitochondria. The acrosome of fresh sperm exhibited a uniform cone-shaped structure with a homogeneous integral composition. The boundary was clearly defined, and the acrosome was slightly compressed. Conversely, the thawing process showed a large gap between the acrosome and the sperm membrane [112]. The alterations in sperm structure may be attributed to various factors, including ice crystal formation, oxidative stress, heat shock, and osmotic shock [73]. Poultry sperm have less cytoplasm and mitochondria but a higher quantity of polyunsaturated fatty acids in the plasma membrane due to water crystallization that occurs during cryopreservation [113]. This distinctive feature is more prone to damage during freezing. This heightened susceptibility is attributed to an increased likelihood of plasma membrane damage caused by water crystallization during cryopreservation [114]. The freezing process disrupts the balance between cellular antioxidant defense systems and the generation of reactive oxygen species (ROS), leading to oxidative stress. This imbalance is often cited as the primary cause of sperm damage during cryopreservation [115]. ROS-induced changes can significantly impact sperm viability and function. Adding enzymatic and non-enzymatic antioxidants can help mitigate negative effects. The freezing process disturbs the equilibrium between ROS production and the sperm’s antioxidant systems, leading to oxidative stress and cellular damage. Supplementing freezing media with antioxidants, such as resveratrol-encapsulated nanoparticles, can safeguard sperm throughout the freezing and thawing process [112].

## 5. Evaluation of Preserved Semen

The evaluation of preserved semen quality involves several parameters, including post-thaw motility, plasma membrane functionality, lipid peroxidation, and mitochondrial activity.

### 5.1. Post-Thaw Motility and Viability

#### 5.1.1. Motility Parameters

Thawed sperm samples were diluted in PBS and placed on a prewarmed slide. Sperm samples were evaluated using SCA software, version 5.1. This computer-assisted sperm analysis determines several parameters, including total motility (TM%), average path velocity (VAP), straight-line velocity (VSL), amplitude of lateral head displacement (ALH), straightness (STR%), linearity (LIN%), and curvilinear velocity (VCL). It can also assess the motion characteristics of at least 400 sperm cells across six fields [116]. The evaluation of preserved semen quality depends on certain standards for motility metrics: a minimum of 40% total motility is accepted as indicative of fertility potential, and reference ranges for VAP, VSL. ALH, STR, LIN, and VCL must be created for reliable assessments [117].

#### 5.1.2. Plasma Membrane Functionality

The integrity of the sperm plasma membrane is a crucial indicator of semen quality, as it plays a vital role in fertilization. Plasma membrane functionality was assessed using the hypo-osmotic swelling test. In this test, sperm are exposed to a hypo-osmotic solution, and sperm with intact functional membranes swell, displaying coiled or swollen tails, indicating their viability. In the experiment, 5 mL of sperm suspension was mixed with 50 mL of a hypo-osmotic solution containing 57.6 mM fructose and 19.2 mM sodium citrate, yielding an osmolarity of 100 mOsm/L. The mixture was incubated at 37 °C for 20 min to assess membrane integrity. The percentage of sperm exhibiting coiled or swollen tails was recorded, as this behavior indicates functional plasma membranes [118]. A threshold of 50% of sperm showing swelling is commonly used to indicate good membrane integrity [119].

#### 5.1.3. Lipid Peroxidation Malondialdehyde (MDA)

Lipid peroxidation is a significant marker of oxidative stress in sperm, often leading to membrane damage and decreased sperm functionality. To evaluate lipid peroxidation, the concentration of malondialdehyde (MDA), a byproduct of lipid oxidation, was measured using a spectrophotometric method. For the analysis, 1 mL of diluted semen containing 250 × 10^6^ spermatozoa was mixed with 1 mL of cold 20% trichloroacetic acid (TCA) to precipitate proteins. The precipitate was then centrifuged at 960× *g* for 15 min. After removing the supernatant, 1 mL was incubated with 1 mL of 0.67% thiobarbituric acid (TBA) in a 95 °C water bath for 10 min. The absorbance at 523 nm was measured using a spectrophotometer, and the MDA concentration was quantified as a measure of lipid peroxidation [120]. Optimal sperm viability is associated with MDA levels lower than 2 nmol/mL [121].

#### 5.1.4. Mitochondrial Activity

Mitochondrial activity plays a crucial role in sperm function, particularly during cryopreservation, as mitochondria generate adenosine triphosphate (ATP), which is vital for sperm motility. Although mitochondrial activity is key to sperm viability, further experimental details on assessing mitochondrial function (such as ATP measurement or mitochondrial integrity assays) are needed to clarify this process, particularly in relation to cryopreserved sperm [60]. During cryopreservation, sperm undergo oxidative stress, leading to the formation of reactive oxygen species (ROS), which can damage the mitochondrial membrane and impair ATP production. This damage results in decreased sperm motility and vitality post-thaw.

Assessing Mitochondrial Activity in Sperm:

- To evaluate mitochondrial activity, various assays are used to measure mitochondrial membrane potential, ATP production, and mitochondrial DNA integrity. Common techniques include the following:
- Mitochondrial membrane potential (Δψm): The mitochondrial membrane potential is a key indicator of mitochondrial health. The JC-1 assay is widely used, where healthy mitochondria accumulate the JC-1 dye and emit red fluorescence, while depolarized mitochondria emit green fluorescence.
- ATP measurement: ATP levels can be measured using bioluminescence-based assays, like the luciferase reaction. Decreased ATP levels are indicative of mitochondrial dysfunction.
- Mitochondrial DNA integrity: the integrity of mitochondrial DNA can be assessed using PCR-based techniques or by measuring oxidative damage markers.
- Recent Studies on Mitochondrial Activity and Cryopreservation:
- Mitochondria-Targeted Antioxidants:
  Astudy investigated the use of mitochondria-targeted antioxidants, such as MitoQ, to mitigate mitochondrial dysfunction and oxidative stress during sperm cryopreservation. The addition of MitoQ at a concentration of 0.02 μM improved post-thaw sperm motility, plasma membrane integrity, and sustained sperm motility for a longer duration. MitoQ supplementation prevented the significant reduction in mitochondrial membrane potential and reduced superoxide production, resulting in lower lipid peroxidation of the sperm plasma membrane after cryopreservation [122].
- Mitochondrial Bioenergetics:
  Another study evaluated the bioenergetic map of mitochondrial metabolism in cryopreserved bovine sperm. The research highlighted the impact of cryopreservation on mitochondrial function and its subsequent effect on sperm motility and viability [123].
- Antioxidant Supplementation:
  Research has also explored the effects of antioxidant supplementation, such as MitoTEMPO, before cryopreservation. MitoTEMPO has been reported to maintain mitochondrial function and viability during the freezing–thawing process through the inhibition of mitochondrial ROS production [124].

## 6. Applications of Poultry Semen Preservation

### 6.1. Conservation of Rare Breeds

Maintaining the genetic resources of farm animals is a critical challenge for preserving domestic biodiversity and supporting the adaption of animal species to global changes, breeding anomalies, or disease epidemics [63]. The genetic material of local or endangered breeds and species with small populations can be preserved in cryogenically stored reproductive cells [6,125,126,127]. For breeding purposes, the cryopreservation of reproductive cells serves as a safeguard for genetic resources and can be a valuable tool for quantifying genetic advancement. Livestock conservation practices have advanced over the past fifty years, leading to the development of new methods for preserving reproductive cells in cryogenic banks. In the last decade, several programs for the ex situ in vitro preservation of endangered breeds through reproductive cell storage has been set up across Europe, North America, Africa, and Asia [6]. Cryobanks now house an array of reproductive cell types, most notably semen and embryos in mammals, semen and primordial germ cells for birds, semen for fish, and larvae for shellfish. Some cryobanks also store somatic cells, driven by the potential to reprogram these cells into viable reproductive cells in the future. The national cryobank of domestic animals in France preserves somatic tissues from birds, mammals, fish, and shellfish, which are conserved in the form of semen, embryos, or larvae depending on the animal (http://www.cryobanque.org/ (accessed on 6 Januray 2025)). Advancements in reproductive physiology and biotechnological capabilities have broadened the diversity of species that can be cryopreserved. Yet, numerous cryobanks are employed merely for cell storage without serving to distribute resources. Notably, the reproductive capacity of cells held in cryobanks is commonly left unassessed [5]. For avian species, semen conservation is the most widely used method for the ex situ in vitro conservation of avian genetic resources and rare breed preservation, as it is the only non-invasive and cost-effective technique available. The cryopreservation of poultry semen, involving cooling, freezing, and thawing, can expose the cells to osmotic and thermal shocks that may harm their structure and metabolism. Extensive research has aimed at determining the best freezing conditions for poultry semen to prevent cell damage and maintain fertilization competence. Crucial elements include the selection of internal and external cryoprotectants, as well as optimizing the cooling and thawing rates, freezing methods, and straw/pellet packaging. The success of freezing protocols can vary significantly based on factors such as genetic line, management conditions, and the specific expertise of each laboratory. This has resulted in the emergence of many different methods. However, these diverse protocols are not commonly used in poultry breeding and still require standardization and demonstration of their efficiency in restoring lost genetic material [75].

Frozen semen is used in situations where AI has been extensively practiced and recognized over a long period. The conception rate with frozen semen is comparable to that of fresh liquid semen. Liquid nitrogen is inexpensive, readily available, and semen containers and instruments are easily accessible. The use of frozen semen leads to enhanced economic benefits [128]. The success of AI techniques in poultry production depends on three crucial elements, namely high-quality semen, proper dilution with compatible diluent solutions, and short-term storage at low temperatures. Diluting the scarce volume of good quality poultry semen is necessary to enable more efficient and economical AI techniques. Furthermore, the utilization of suitable diluents is paramount, as they help in maintaining the viability and fertilizing potential of spermatozoa during storage. Genetic conservation methodologies include AI, semen gonad storage, and primordial germ cells (PGCs). These have been widely employed in the breeding management of various poultry species, including chickens (*Gallus gallus domesticus*), quails (*Coturnix coturnix*), and turkeys (*Meleagris gallapavo*). These methodologies are poised to play an increasingly important role in ensuring the sustainability, resilience, and innovation of poultry production systems.

### 6.2. Artificial Insemination

AI is considered an important tool for the poultry industry. Using frozen semen allows for the effective use of male birds, which is not always feasible under natural mating conditions. This directly reduces the demand for cockerels in gamete provision, leading to a decrease in the overall cost of poultry production. AI was the initial biotechnological tool employed to boost poultry production, as it enabled the use of genetically superior cockerels demonstrating high levels of productive performance [129]. The first steps to introduce AI as a feasible procedure began in Russia in 1899 under Ivanov, who studied its application in domestic farm animals, including poultry [130]. AI in birds was first successfully demonstrated nearly a century ago, when Ivanov produced fertile chicken eggs using semen extracted from the ductus deferens of a cockerel [131]. In chickens, the collection of artificial semen was facilitated by devices developed by Ishikawa [132]. In 1935, Burrows and Quinn introduced an abdominal massage technique for chickens, which was refined and came to be known as “milking the male” [133]. The method for semen collection followed by AI was then developed by Quinn and Burrows [134]. Consequently, these researchers are considered the founders of avian AI, and their fundamental technique is still employed across various poultry species. The use of AI became widespread with the introduction of laying cages in Israel [135] and Australia [136]. Concurrently, in the USA, AI was employed to enhance the fertility of broiler chickens. In India, this technique was incorporated into poultry farming with the establishment of the All India Coordinated Research Project on Poultry in 1957. AI is commonly employed with freshly collected semen due to the ease of collection and the proximity of hens in large breeding operations for insemination. Since the 1960s, the AI technique has emerged as a pivotal component of reproduction in turkeys and is used almost exclusively for commercial flock management. The significant size differences between male and female turkeys lead to ineffective natural mating low fertilization rates in heavy broad-breasted varieties, making AI essential for commercial turkey farming [38].

AI is gaining momentum in other species as well. For example, as fertility declines in broiler breeds due to selective breeding for growth, AI may become a cost-effective solution in broiler breeder management [137]. However, the application of AI faces challenges in species such as quail and guinea fowl, attributed to the presence of cloacal gland foam and low semen volume [138,139,140]. Similarly, the integration of AI is not straightforward in ducks (*Anas platyrhynchos*) and geese (*Anser anser domesticus*), as their oviducts cannot be everted like those in chickens (*Gallus gallus domesticus*) and turkeys (*Meleagris gallopavo*), resulting in limited commercial demand for AI in these species [50]. Conversely, AI techniques have been successfully adapted for use in cranes (*Grus*) and other wild birds, which is pivotal for the preservation of endangered species and the establishment of viable self-perpetuating populations [141,142].

## 7. Future Directions and Research Needs

The poultry industry must address challenges related to genetic diversity and production efficiency. To tackle this issue, enhancing semen preservation methods is vital. Future research should focus on developing better cryoprotectants to prevent cellular damage during freezing and thawing processes. Gaining insights into the mechanisms of semen damage will support targeted solutions. Investigating novel preservation methods, such as vitrification, along with assessing extender composition, is also essential. Additionally, integrating biotechnological innovations, such as CRISPR, can lead to improved semen quality and enhance disease resistance. Addressing these needs will further promote both effective preservation and sustainable practices in poultry.

## 8. Conclusions

In conclusion, preserving poultry semen is crucial for ensuring genetic diversity and improving production efficiency in the poultry sector. The research field is dynamic, with impressive advancements. Ongoing investigations are necessary, particularly in creating effective cryoprotectants and optimizing preservation techniques. Moreover, applying modern technologies such as omics can deepen our understanding of the factors influencing semen quality and viability. By revealing the underlying mechanisms of semen preservation, we can formulate new strategies to enhance preservation efficiency, thereby supporting poultry production and promoting the welfare of poultry species.

## Figures and Tables

**Table 2:** Composition of different extenders used for mid-term poultry semen storage (30 min–48 h) [80].

Item	L&R-84 pH = 7.08 343 mOsm/kg	Lake 7.1 pH = 7.1 370 mOsm/kg	EK media pH = 7.8 390 mOsm/kg	BPSE pH = 7.5 333 mOsm/kg	ASG pH = 7.1 325 mOsm/kg
**Sodium-L-glutamate**	19.2 g	15.2 g	14 g	8.76 g	12.11 g
**Glucose**	8 g	6.0 g	7 g		5.26 g
**Magnesium acetate 4 H_2_O**	0.8 g	0.8 g			0.64 g
**Potassium acetate**	5.0 g				
**Polyvinyl pyrrolidone**	3.0 g		1 g		
**Potassium citrate tribasic 1 H_2_O**		1.28 g	1.4 g	0.6 g	1.02 g
**D-fructose**			2 g	5.0 g	
**TES**				1.95 g	
**Sodium acetate 3 H₂O**				4.3 g	
**Sodium hydroxide 1 N**		58 mL			1.85 mL
**Sodium dihydrogen phosphate**			2.1 g		
**Disodium hydrogen phosphate**			9.8 g		
**BES**		30.5 g			24.3 g
**Potassium diphosphate 3 H_2_O**				12.7 g	
**Potassium monophosphate**				0.65 g	
**Magnesium chloride**				0.34 g	
**Inositol**			7 g		
**Protamine sulfate**			0.2 g		
**H_2_O**	1000 mL	1000 mL	1000 mL	1000 mL	100 mL

## Data Availability

Data generated or analyzed during this study will be made available upon request to the corresponding author.
